# Potential application of pig (*Sus scrofa domestica*) skin peptide-iron chelates in the treatment of iron deficiency anemia and regulation of intestinal flora metabolism

**DOI:** 10.3389/fnut.2025.1553668

**Published:** 2025-06-13

**Authors:** Shuteng Huang, Hanxiu Deng, Kexin Liang, Xue Zhao, Jiayu Zhang

**Affiliations:** ^1^School of Pharmacy, Binzhou Medical University, Yantai, China; ^2^Yantai Marine Key Laboratory, Yantai, China; ^3^School of Pharmacy, Shandong University of Traditional Chinese Medicine, Jinan, China

**Keywords:** pig (*Sus scrofa domestica*) skin, iron deficiency anemia, peptide-iron chelates, iron supplementation, gut microbiota

## Abstract

Iron deficiency is an important public health concern worldwide. Intake of iron-fortified foods has been widely used to treat iron deficiency anemia (IDA). In this study, a novel food for iron supplementation was designed: pig (*Sus scrofa domestica*) skin peptide-iron (PSP-Fe) chelates. Structural characterization demonstrated that acidic amino acids (aspartic acid, glutamic acid) and aromatic amino acids (phenylalanine, tryptophan, and tyrosine) in PSP were involved in the chelation reaction, with the carboxyl group and amino group provided the major iron binding sites. In addition, iron significantly altered the microscopic morphology of PSP. IDA rats were established and different doses of iron supplements were gavaged for 21 days to evaluate the effectiveness of PSP in treating IDA. The medium dose of PSP-Fe restored hemoglobin (HGB), red blood cell (RBC), hematocrit (HCT), mean corpuscular hemoglobin concentration (MCHC), serum ferritin (SF), serum iron (SI), hepcidin, total iron binding capacity (TIBC) and transferrin saturation (TSAT) to normal levels. PSP-Fe also ameliorated the abnormal changes in heart coefficients, lungs coefficients, liver coefficients and spleen coefficients caused by IDA. PSP-Fe further restored iron storage in the liver and villous damage in the colon of rats compared to FeSO_4_. 16S rRNA results suggest that the 10 microbial markers in the Model group may impede iron absorption and HGB synthesis of host through biosynthesis of siderophore group nonribosomal peptides, vitamin B6 metabolism, lipoic acid metabolism, ascorbate metabolism and tryptophan metabolism. At the end, the safety of PSP-Fe was preliminarily affirmed by toxicity evaluation *in vitro* and *in vivo*. These findings suggest that PSP-Fe has potential as a novel functional food for treating IDA.

## Graphical abstract

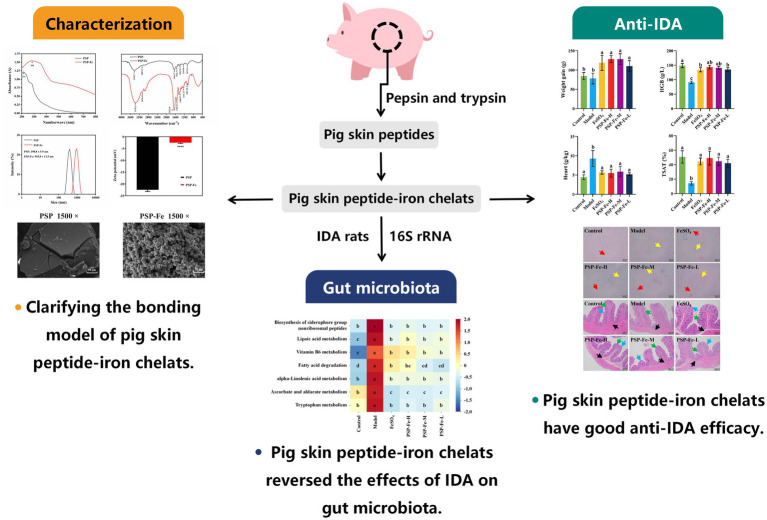

## 1 Introduction

Iron is a trace element necessary for normal functioning of the human body as it is involved in basic biochemical processes such as transport of oxygen, electron transfer, DNA synthesis, and cell differentiation (1–3). The physiological range of iron content in adult’ body is 38–50 mg/kg, and most of the iron (>70%) is present in hemoglobin (HGB) (4, 5). Iron deficiency (ID) is an important public health problem worldwide with a much more serious in developing countries than in developed countries, especially in areas where nutritious diverse diets are not available or affordable (6). Iron deficiency anemia (IDA) is one of the most common nutritional disorders globally, affecting >2 billion individuals, with a high prevalence in children <5 years of age, women of child-bearing age, and pregnant women (7, 8). IDA is mainly caused by a low iron intake (lack of iron-rich foods or other reasons for not absorbing iron properly), excessive iron loss (chronic gastrointestinal bleeding), and an increased iron demand (children at the stage of growth) (9). ID can cause developmental delays and cognitive impairment in young children (10). Iron imbalances in the human brain also increase the risk of neurodegenerative diseases (11). Furthermore, ID during pregnancy may lead to low birth weight and preterm delivery of the offspring (12). Therefore, prevention and effective treatment of ID is critical for these populations.

Intake of iron-fortified foods has been widely used to treat IDA worldwide. Most of the oral supplements currently available in the market, such as ferrous sulfate (FeSO_4_), ferrous lactate, ferrous glycinate, and ferrous gluconate, have strong gastrointestinal adverse effects, low bioavailability, poor patient compliance, and relatively high preparation costs (9, 13). Therefore, the development of a novel functional food for iron supplementation that is safe, effective, and economical is imminent.

As one of the dietary supplements to alleviate IDA, peptide-iron chelates have several advantages, including high bioavailability, good stability, and no adverse effects (14, 15). Currently, there are three main transport pathways by which chelates promote the absorption of iron: the ferrous ion transport pathway, the peptide absorption pathway, and peptides as metal ion transport carriers (16). In addition, chelates can prevent ferrous iron from forming insoluble complexes with phytic acid, polyphenols (e.g., tannins), and oxalic acid (17, 18). Chelates can substantially increase the absorption of iron, and iron-chelating active peptides can be obtained from different food or by-product, including oysters (19), tilapia skin (20), Alaskan pollock skin (21), whey (22), oats (23), and mung beans (24).

Pig skin is an economic by-product of pig processing. At present, pig farming is accelerating worldwide with the rapid expansion of industrial farming. In 2021, pork accounted for 34% of the global meat production, with >1.4 billion commercial pigs in slaughterhouses (25). The rich proteins contained in the dermis of pig skin may be an excellent source of ion chelating peptides; however, the study of pig skin peptide-iron (PSP-Fe) chelates has received little attention.

The aim of our study was to elucidate the binding mode of PSP to iron and to assess the efficacy of PSP-Fe in the treatment of IDA. In addition, the pathways of microbial markers affecting host hemoglobin synthesis were further screened and explored. These results provide references and bases for the comprehensive utilization of pig skin and the development of new functional food for iron supplementation.

## 2 Materials and methods

### 2.1 Materials and reagents

Pig skins were purchased from Yantai Xiwang Meat Products Co., Ltd. (Yantai, China). Pepsin (2,500 units/mg), trypsin (2,500 units/mg), FeCl_2_·4H_2_O, ascorbic acid, phenanthroline, sodium acetate buffer, hydroxylamine hydrochloride, KBr and anhydrous ethanol were purchased from Shanghai Aladdin Biochemical Science Technology Co., Ltd. (Shanghai, China). UHPLC grade acetonitrile, methanol and formic acid (FA) were purchased from Thermo Fisher Scientific (Fair Lawn, NJ, United States), and pure water for analysis was bought from Watson Group Co., Ltd. (Guangzhou, China).

### 2.2 Preparation of PSP

Fresh pig skins were degreased with petroleum ether and then were placed in distilled water (12.5 g PSP per 100 mL). Subsequently, the proteins were subjected to sequential enzymatic hydrolysis with pepsin (enzyme-substrate mass ratio 1:100, pH 1.5, 1 h) and trypsin (enzyme-substrate mass ratio 1:100, pH 8.0, 3 h) at 37°C. Immediately after hydrolysis, the reaction mixture was heated at 95°C for 10 min to inactivate the enzyme, and then centrifuged at 5,000 rpm for 10 min. The supernatant was ultrafiltered using a 3 kDa cut-off membrane and the filtrate was lyophilized to obtain PSP powder.

### 2.3 Preparation of PSP-Fe chelates

The PSP-Fe was prepared using the method proposed by Zhao et al. (26) with some modifications. The PSP was dissolved in distilled water (3 g PSP per 10 mL), ascorbic acid was added at a mass ratio of 0.4:1 of ascorbic acid to FeCl_2_·4H_2_O, and the pH was adjusted to 5.0. Subsequently, FeCl_2_·4H_2_O was added at a mass ratio of 10:1 of PSP to FeCl_2_·4H_2_O, and stirred for 40 min at 40°C. Immediately after the reaction, seven-fold anhydrous ethanol was added (volume/volume, v/v) to remove peptides not involved in the reaction. The mixture was centrifuged at 5,000 rpm for 10 min and the precipitate was lyophilized to obtain PSP-Fe.

Iron content was determined with reference to the method described by Wu et al. (27) with some modifications. PSP-Fe was dissolved in distilled water to prepare a 1 mg/mL solution. Then, 10 mL PSP-Fe solution, sodium acetate buffer, 10 mg/mL hydroxylamine hydrochloride, 1 mg/mL phenanthroline and distilled water were added sequentially into a 25 mL volumetric flask. The absorbance was recorded by an ultraviolet–visible (UV–Vis) spectrophotometer (UV-3600i Plus, Shimadzu, Japan) at 510 nm.

### 2.4 Characterization of the PSP and PSP-Fe chelates

#### 2.4.1 UV–Vis absorption spectroscopy

The PSP or PSP-Fe was dissolved in distilled water to prepare a 0.1 mg/mL solution. The absorption spectra of PSP or PSP-Fe was measured in the wavelength range of 200–800 nm by the UV–Vis spectrophotometer.

#### 2.4.2 Fourier-transform infrared spectroscopy

The PSP or PSP-Fe was mixed with 90 mg of dry KBr, thoroughly ground several times, and then pressed into transparent flakes. The Fourier-transform infrared (FTIR) spectra of PSP or PSP-Fe was obtained by a FTIR spectrometer (Nicolet iS50, Thermo Fisher Scientific, United States) in the wave number range from 4,000 cm^−1^ to 400 cm^−1^.

#### 2.4.3 Particle size and zeta potential

The PSP or PSP-Fe was dissolved in distilled water to prepare a 1.5 mg/mL solution. The particle size and zeta potential of PSP or PSP-Fe was obtained by a laser particle size analyzer (Mastersizer 3000, Malvern Panalytical, United Kingdom), and the samples were tested three times.

#### 2.4.4 Scanning electron microscopy analysis

An appropriate amount of PSP or PSP-Fe was evenly spread on the tapes on the sample rack with vacuum sprayed gold treatment, and the microstructure of the samples was observed by scanning electron microscopy (SEM) (EVO LS15, ZEISS, Germany). The magnification was 1,500 and 5,000, respectively.

#### 2.4.5 Amino acid composition analysis

The PSP or PSP-Fe (100 mg) was hydrolyzed with 15 mL of 6 M HCl in a sealed hydrolysis tube under reduced pressure at 110°C for 24 h. Hydrolyzed samples were filtered, dried, and solubilized, and the amino acid composition was determined using an amino acid analyzer (Biochrom30+, Biochrom, United Kingdom).

#### 2.4.6 High-performance liquid chromatography (HPLC) mass spectrometry (MS)/MS analysis

The peptide sequences of PSP-Fe were detected using EASY-nLC1200 Q Exactive Plus (Thermo Scientific, United States). The samples were desalted, dried, and dissolved, and separated on a C18 reversed-phase analytical column (75 mm × 20 cm × 3 μm). Mobile phase A was a mixture of 99.9% water and 0.1% FA, and mobile phase B was a mixture of 80% acetonitrile and 0.1% FA. The liquid phase gradients were as follows: 0–3 min, 2–6% B; 3–42 min, 6–20% B; 42–47 min, 20–32% B; 47–48 min, 32–100% B; 48–60 min, 100% B. The mobile phase flow rate was 300 nL/min. ESI^+^ assay mode was used in a data-dependent scanning mode at a resolution of 70,000 orbital trap for full-scan acquisition. An induced collision-induced dissociation MS/MS scan was then performed. Daughter ions were measured in orbitals with a resolution of 17,500. Finally, peptide sequences were obtained using software analysis and protein database comparison.

### 2.5 Efficacy of PSP-Fe in the treatment of IDA

#### 2.5.1 Animals, diets and treatment

Forty-two 3-week-old male Sprague–Dawley rats (45–50 g) were purchased from Jinan Pengyue Laboratory Animal Breeding Co., Ltd. [License No. SCXK (Lu) 2022-0006] (Jinan, China). AIN-93G standard diet (Fe = 50 mg/kg) and modified AIN-93G ID diet (5 mg Fe per kg) were purchased from Beijing Keao Xieli Animal Feed Co., Ltd. (Beijing, China). All rats were housed at standard environmental conditions (24 ± 2°C, 50 ± 5% humidity) with free access to water. Before starting the experiments, rats were allowed to acclimatize to the environment for 3 days and were fed standard diet and deionized water.

After 3 days of acclimatization, seven rats were randomly selected as the control group and fed with standard diet. Thirty-five rats were fed with ID diet for 28 days. After 2 weeks of feeding, blood was collected weekly from the tail vein and the HGB level was monitored using an animal hematology analyzer. IDA was defined when the HGB level was lower than 100 g/L.

After IDA rats were established, the control group continued to be given a standard diet. Thirty-five IDA rats were divided into five groups (Model, FeSO_4_, PSP-Fe-H, PSP-Fe-M, and PSP-Fe-L) of seven rats each and fed the ID diet. Each group was gavaged with deionized water [10 mL/kg·body weight (bw)] or the corresponding iron supplement (dissolved in deionized water, 10 mL/kg·bw) once daily at 9:00 a.m. for 21 days. The dosages for each group were as follows: Control and Model groups (deionized water), FeSO_4_ group (Fe = 2 mg/kg·bw), PSP-Fe-H group (Fe = 3 mg/kg·bw), PSP-Fe-M group (Fe = 2 mg/kg·bw), and PSP-Fe-L group (Fe = 1 mg/kg·bw).

#### 2.5.2 Sample collection

All rats were weighed weekly. At the end of the experiment, all rats were fasted for 12 h, then anesthetized with 2% sodium pentobarbital. Blood samples were collected from the abdominal aorta, and serum was separated by centrifugation at 3,000 rpm for 6 min. The heart, liver, spleen, lungs, and kidney organs were removed, rinsed, blotted and weighed. Liver and colon were stored in 4% paraformaldehyde solution. Cecal contents were collected, placed in freezing tubes, and stored at −80°C.

#### 2.5.3 Hematological tests

Hematological parameters were obtained by an animal hematology analyzer, including red blood cell (RBC), hematocrit (HCT), mean corpuscular volume (MCV), mean corpuscular hemoglobin (MCH), mean corpuscular hemoglobin concentration (MCHC), and red blood cell distribution width (RDW). Serum ferritin (SF), serum iron (SI) and total iron binding capacity (TIBC) were obtained by a biochemical analyzer. Hepcidin was measured with an ELISA kit (Shanghai Enzyme-linked Biotechnology Co., Ltd., Shanghai, China). Transferrin saturation (TSAT) was calculated as follows:


TSAT(%)=SI(μmol/L)/TIBC(μmol/L)×100%

#### 2.5.4 Organ coefficient

The relative weight indices of the heart, liver, spleen, lungs and kidney were calculated from the final body weight measured at the end of the experiment. The organ coefficients were calculated as follows:


Organ coefficient(g/kg)=Organ weight(g)/Ratweight(kg)

#### 2.5.5 Histological analysis

The colonic tissues were fixed in 4% paraformaldehyde solution, and the fixed tissue blocks were dehydrated stepwise, transparent with xylene, impregnated in wax and embedded in paraffin in order to prepare 5 μm-thick sections for hematoxylin and eosin (H&E) staining. They were examined using a biomicroscope at magnification of 100. Liver was stained using Prussian blue as described above to visualize iron storage in the liver.

#### 2.5.6 High-throughput sequencing analysis

Microbiota analysis was performed on the Illumina NoveSeq 6000 sequencing platform (Illumina, San Diego, CA, United States). Primers 341F and 806R were used to amplify the V3 + V4 variable region of the 16S rRNA gene. Sequencing libraries were generated by NEB Next Ultra DNA Library Prep Kit (Illumina, San Diego, CA, United States), and the libraries were tested using a bioanalyzer (Agilent 5400, Agilent Technologies Co., Ltd., United States) and PCR quantification (T100PCR, Bio-Rad, United States). Once the libraries were qualified, up-sequencing was performed using the Illumina sequencing platform and 250 bp paired-end reads were generated. The Greengenes 2 database was used as the reference dataset. Raw sequences from each sample were quality filtered, and then the chimeric sequences were identified and removed by the plug-in in the QIIME2 software to obtain the operational taxonomic units (OTUs). Metabolic pathway enrichment analysis was performed by the Kyoto Encyclopedia of Genes and Genomes (KEGG) (28).

### 2.6 Toxicity evaluation of PSP-Fe *in vitro*

#### 2.6.1 Cell culture

Human renal proximal tubular epithelial cells (HK-2) were purchased from Wuhan Pricella Biotechnology Co., Ltd. (Wuhan, China) and cultured in MEM (Pricella, Wuhan, China) supplemented with 10% fetal bovine serum (Pricella, Wuhan, China) and 1% penicillin-streptomycin (Gibco, United States). HK-2 cells were cultured in a 37°C and 5% CO_2_ incubator under a humidified atmosphere.

#### 2.6.2 CCK-8 assay

HK-2 cells were seeded in 96-well plates at a concentration of 5 × 10^3^ cells/mL. After overnight culture, medium containing different concentrations of PSP-Fe (Fe = 0, 31, 63, 125 and 250 μg/mL) was added and cultured for 24 h. Then, 10 μL CCK-8 solution (Beijing Lablead Biotechnology Co., Ltd., Beijing, China) was added to each well and cultured at 37°C for 3 h. Absorbance values of the solution were detected using a microplate reader (Thermo Scientific, United States) at 450 nm. Cell viability was calculated as follows:


$$\begin{array}{l} \text{Cell viability}\;(\%)=(\begin{array}{l} \text{OD}\;\text{of experimental group}-\\ \text{OD}\;\text{of blank group} \end{array})/\\ (\begin{array}{l} \text{OD}\;\text{of control group}-\\ \text{OD}\;\text{of blank group} \end{array})\times 100\% \end{array}$$

### 2.7 Toxicity evaluation of PSP-Fe *in vivo*

#### 2.7.1 Animals and treatment

Twenty-eight 6-week-old male Sprague–Dawley rats (190–200 g) were purchased from Jinan Pengyue Laboratory Animal Breeding Co., Ltd. AIN-93G standard diet were purchased from Beijing Keao Xieli Animal Feed Co., Ltd. All rats were housed at standard environmental conditions with free access to water. Before starting the experiments, rats were allowed to acclimatize to the environment for 3 days and were fed standard diet and deionized water. After 3 days of acclimatization, all rats were randomly divided into four groups (Control, PSP-Fe-H, PSP-Fe-M, and PSP-Fe-L) of seven rats each and fed the standard diet. Each group was gavaged with deionized water (10 mL/kg·bw) or the corresponding iron supplement (dissolved in deionized water, 10 mL/kg·bw) once daily for 28 days. The dosages for each group were as follows: Control groups (deionized water), PSP-Fe-H group (Fe = 3 mg/kg·bw), PSP-Fe-M group (Fe = 2 mg/kg·bw), and PSP-Fe-L group (Fe = 1 mg/kg·bw).

#### 2.7.2 Sample collection

All rats were weighed weekly. At the end of the experiment, all rats were fasted for 12 h, then anesthetized with 2% sodium pentobarbital. Blood samples were collected from the abdominal aorta, and serum was separated by centrifugation at 3,000 rpm for 6 min. The heart, liver, spleen, lungs, and kidney organs were removed, rinsed, blotted, weighed and stored in 4% paraformaldehyde solution.

#### 2.7.3 Hematological tests

Serum alanine aminotransferase (ALT), aspartate aminotransferase (AST), blood urea nitrogen (BUN) and creatinine (CR) were obtained by a biochemical analyzer. Serum superoxide dismutase (SOD) and glutathione (GSH) were measured with corresponding assay kits (Wuhan Servicebio Technology Co., Ltd., Wuhan, China).

#### 2.7.4 Organ coefficient

The relative weight indices of the heart, liver, spleen, lungs and kidney were calculated from the final body weight measured at the end of the experiment.

#### 2.7.5 Histological analysis

The heart, liver, spleen, lungs, and kidney tissues were fixed in 4% paraformaldehyde solution, and the fixed tissue blocks were dehydrated stepwise, transparent with xylene, impregnated in wax and embedded in paraffin in order to prepare sections for H&E staining. They were examined using a biomicroscope at magnification of 100.

### 2.8 Statistical analysis

All data were presented as mean ± standard deviation. Statistical analyses were assessed by Student’s *t*-test, one-way ANOVA and Duncan’s multiple range method, statistically significant differences between the groups were considered as *p* < 0.05.

## 3 Results

### 3.1 Characterization of PSP and PSP-Fe chelates

#### 3.1.1 UV–Vis absorption spectroscopy

**Figure 1:**
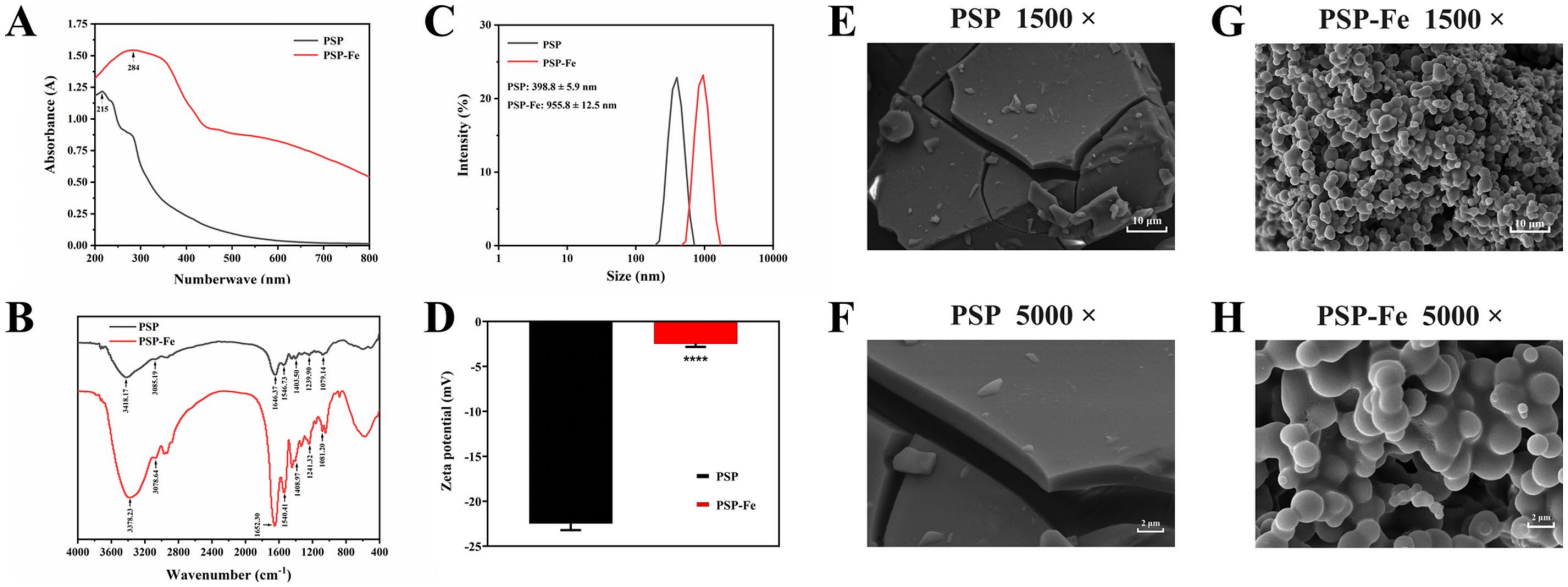
Structural characterization of PSP and PSP-Fe. **(A)** UV–Vis spectrum. **(B)** FTIR spectrum. **(C)** Particle size distribution. **(D)** Zeta potential. Microstructures of PSP at magnifications of **(E)** 1,500 and **(F)** 5,000. Microstructures of PSP at magnifications of **(G)** 1,500 and **(H)** 5,000. ^****^p < 0.0001 versus the PSP.

As shown in Figure 1A, the maximum UV–Vis absorption peak of PSP shifted from 215 nm to 284 nm after chelating with iron. This suggests that aromatic amino acids, such as phenylalanine, tryptophan, and tyrosine, may be involved in the chelation of PSP with iron (15). In addition, the absorption peak strength of the ferrous ions of the ring of PSP-Fe was higher than that of PSP due to the valence electron transition. Zhao et al. (29) concluded that upon combining a peptide with a metal ion, the chirality of the chromophore changes, including C=O, −COOH, −OH, and −NH_2_, resulting in a change in the spectrum. A similar variation in the UV–Vis spectra of the tilapia skin collagen iron-chelating peptides was observed (20).

#### 3.1.2 FTIR spectroscopy

When ferrous ions chelate with the amino acid residues of PSP, clear shifts in the absorption peaks can be observed in the FTIR spectrum. As shown in Figure 1B, the absorption peak of PSP at 3,418 cm^−1^ corresponds to the stretching vibration of the N–H bond. After chelating ferrous ions, the absorption peak of PSP-Fe was shifted to 3,378 cm^−1^, indicating that the N–H bond of PSP was involved in the chelating reaction. The absorption peaks of PSP at 1,646 cm^−1^ and 1,546 cm^−1^ corresponded to the amide I band and the amide II band, which were generated by the stretching vibration of the C=O bond and the bending vibration of the N–H bond, respectively. The absorption peaks corresponding to PSP-Fe were shifted to 1,652 cm^−1^ and 1,540 cm^−1^, respectively, indicating that the C=O and N–H bonds provide coordination sites for the ferrous ions. The absorption peak of PSP at 1,403 cm^−1^ shifted to 1,408 cm^−1^, indicating that the ferric chelating site is also related to the carboxyl group on the peptide chain. In addition, the absorption peak of PSP-Fe at 1,081 cm^−1^ may be due to the formation of C–O–Fe coordination bonds. Overall, the -NH_2_ and –COOH groups of PSP provided the major binding sites for ferrous ions. Similar results confirmed that silver carp scale collagen peptide binds to iron mainly through amino and carboxylic acid groups (26). Furthermore, Liu et al. (19) reported that ferrous ions could bind to –NH_2_ and –COOH groups of oyster protein hydrolysate to form chelates.

#### 3.1.3 Particle size and zeta potential

The particle size of PSP and PSP-Fe are shown in Figure 1C. The results showed that the particle size of PSP (398.8 ± 5.9 nm) was significantly (*p* < 0.0001) lower than that of PSP-Fe (955.8 ± 12.5 nm). The increase in particle size distribution indicated that the chelating reaction between ferrous ions and peptides exists not only in intramolecular interactions but also in intermolecular interactions (30). A single ferrous ion may bind to multiple peptide chains at the same time. The polydispersity index of PSP and PSP-Fe ranged from 0.279 to 0.313, suggesting an acceptable homogeneity.

The zeta potential is an important indicator of the surface charge state of particles in a dispersed system. As shown in Figure 1D, the zeta potential of PSP increased significantly from −22.5 ± 0.72 mV to −2.49 ± 0.34 mV after chelating with ferrous ions (*p* < 0.0001). This may be due to the binding of ferrous ions to carboxyl groups during chelation neutralizing some of the negative charges. A similar conclusion was reached by Li et al. (31) for the zeta potential result of duck egg white peptide-ferrous chelate.

#### 3.1.4 SEM analysis

SEM can visualize the object micromorphology. The microstructures of PSP and PSP-Fe were observed at magnifications of 1,500 and 5,000, respectively (Figures 1E–H). The surface of PSP was relatively smooth and dense, and was in the form of blocks. Interestingly, PSP-Fe showed loose particle aggregates. The reason for the change in microstructure may be that the carboxyl group of PSP formed a “bridging role” with ferrous ions, destroying the original dense structure of the peptide surface (32). This phenomenon is also similar to that of the tilapia skin collagen iron-chelating peptides (20).

#### 3.1.5 Amino acid composition analysis

Since various amino acids have different binding affinities for iron, changes in amino acid composition are of interest. The amino acid composition and relative content of PSP and PSP-Fe are shown in Table 1. The relative content of aspartic acid and glutamic acid were increased after the chelation of PSP with ferrous ions. Among them, the contents of aspartic acid increased significantly from 50.48 ± 1.05 mg/g to 58.29 ± 0.67 mg/g (*p* < 0.05), and the contents of glutamic acid increased significantly from 83.82 ± 0.25 mg/g to 95.11 ± 0.34 mg/g (*p* < 0.05). This result indicated that the carboxyl groups these two acidic amino acids contributed significantly to the chelation of iron. Through detailed experiments, Hu et al. (33) verified that ferrous iron can bind to the carboxyl group of aspartic acid and the imidazolyl group of histidine in the Antarctic krill heptapeptide through coordinate bonds and *π*-cation interactions, respectively. In addition, Huang et al. (29) isolated a tripeptide with calcium chelating activity in the hydrolysate of shrimp processing byproducts with the sequence “threonine-cysteine-histidine”.

**Table 1:** Amino acid composition of PSP and PSP-Fe.

Amino acids	Relative content (mg/g)	
	PSP	PSP-Fe
Aspartate	50.48 ± 1.05^a^	58.29 ± 0.67^b^
Threonine	16.46 ± 1.41^a^	16.09 ± 0.99^a^
Serine	29.55 ± 0.34^a^	28.81 ± 0.83^a^
Glutamate	83.82 ± 0.25^a^	95.11 ± 0.34^b^
Glycine	116.76 ± 1.67^a^	115.45 ± 1.17^a^
Alanine	67.17 ± 0.10^a^	62.66 ± 0.44^b^
Cysteine	16.10 ± 1.22^a^	16.73 ± 0.56^a^
Valine	22.59 ± 0.38^a^	18.88 ± 1.35^b^
Methionine	9.11 ± 0.14^a^	8.07 ± 0.35^b^
Isoleucine	12.74 ± 0.95^a^	8.43 ± 0.11^b^
Leucine	29.27 ± 0.57^a^	22.52 ± 0.30^b^
Tyrosine	9.70 ± 0.36^a^	9.37 ± 0.84^a^
Phenylalanine	20.70 ± 0.73^a^	18.19 ± 1.52^a^
Histidine	8.36 ± 0.61^a^	8.71 ± 0.30^a^
Lysine	39.44 ± 0.79^a^	38.05 ± 0.61^a^
Arginine	71.08 ± 1.20^a^	69.55 ± 1.07^b^
Proline	104.29 ± 1.23^a^	95.20 ± 1.88^b^

Different letters (a,b) showed significant difference (*p* < 0.05).

#### 3.1.6 HPLC MS/MS analysis

Two enzymes were used in the preparation of PSP. Pepsin tends to hydrolyze peptide bonds with aromatic amino acids at the amino terminus or carboxyl terminus, while the site of action of trypsin is generally after the lysine and arginine, yielding peptides with either lysine or arginine as the carboxyl terminus residue. The molecular weight of peptides is an important factor influencing metal chelating activity. Low molecular weight peptides have better metal chelating activity compared to high molecular weight peptides. Table 2 demonstrates the top 30 peptide sequences with the highest peak areas, of which 17 peptides have molecular weights located in the range of 1–2 kDa, and 10 peptides have molecular weights <1 kDa. Fan et al. (16) identified 17 peptides with iron-chelating activity from hydrolysates of sea cucumber, and their molecular weights were all <2 kDa. Guo et al. (21) identified an iron-chelating decapeptide from Alaska pollock skin; the peptide sequence was GPAGPHGPPG. Huang et al. (34) reported that small molecular weight peptides (<5 kDa) extracted from hairtail protein hydrolysates had better ferrous iron-chelating activity than large molecular weight peptides (>5 kDa).

**Table 2:** Peptide sequence identified in PSP-Fe.

Number	Identified peptide sequence	Molecular weight (Da)	Retention time (min)
1	GQAGVMGFPGPK	1144.5699	21.19
2	GFSGLDGAK	850.4185	16.76
3	GFPGPKGANGEPGK	1311.6571	31.93
4	ISVPGPMGPSGPR	1250.6440	31.42
5	SGDRGETGPAGPAGPVGPVGAR	1960.9714	19.47
6	GYEGDFY	849.3181	30.82
7	GFPGLPGPSGEPGK	1295.6509	37.80
8	GVVGLPGQRGER	1223.6735	15.35
9	SAGISVPGPMGPSGPR	1465.7347	35.45
10	DIASTPHELYR	1300.6411	21.77
11	GVVGLPGQR	881.5083	21.05
12	ISVPGPMGPSGPR	1250.6441	23.58
13	GFSGLDGAKGDAGPAGPK	1600.7844	20.14
14	GETGPAGPAGPVGPVGAR	1545.7899	23.31
15	SHNQMQEHVDLRDPNIR	2087.9919	19.04
16	EGLRGPRGDQGPVGR	1549.8073	9.54
17	DSFQEVLR	992.4927	30.35
18	FGYEGDF	833.3231	43.42
19	AHDGGRYY	937.4042	8.18
20	GHNGLQGLPGLAGHHGDQGAPGPVGPAGPR	2777.3858	33.92
21	SFLPQPPQEK	1169.6080	30.22
22	GHAGLAGAR	808.4304	7.20
23	GSEGPQGVR	885.4304	7.80
24	GEAGPQGAR	841.4042	6.75
25	VGLEHLR	822.4712	13.20
26	SFLPQPPQE	1041.5131	44.36
27	LIDSSDGVKPDGIAHIRD	1906.9749	28.43
28	LYLRNNQIDHIDDK	1755.8904	25.65
29	FSFLPQPPQEK	1316.6764	50.41
30	GYDEKSAGISVPGPMGPSGPR	2057.9839	33.78

Peptides have different binding sites for ferrous ions including terminal amino groups and carboxyl groups, nitrogen atoms of the peptide chain, and active side chains of amino acids (35). Athira et al. (15) classified amino acids that provide active side chains for metal chelation into three categories: non-coordinating, weakly coordinating, and strongly coordinating side chains. Of the 30 peptides we identified, each peptide contained 1–9 coordinating amino acids, which could be the significant reason for the good iron-binding effects of PSP-Fe. In addition, although non-coordinating amino acids do not directly act as iron binding sites, they can provide stability and conformation for peptide-iron chelates through non-covalent interactions, such as hydrophobic interactions, van der Waals forces and hydrogen bonding (35, 36).

### 3.2 Efficacy of PSP-Fe in the treatment of IDA

#### 3.2.1 Changes in body weight

**Figure 2:**
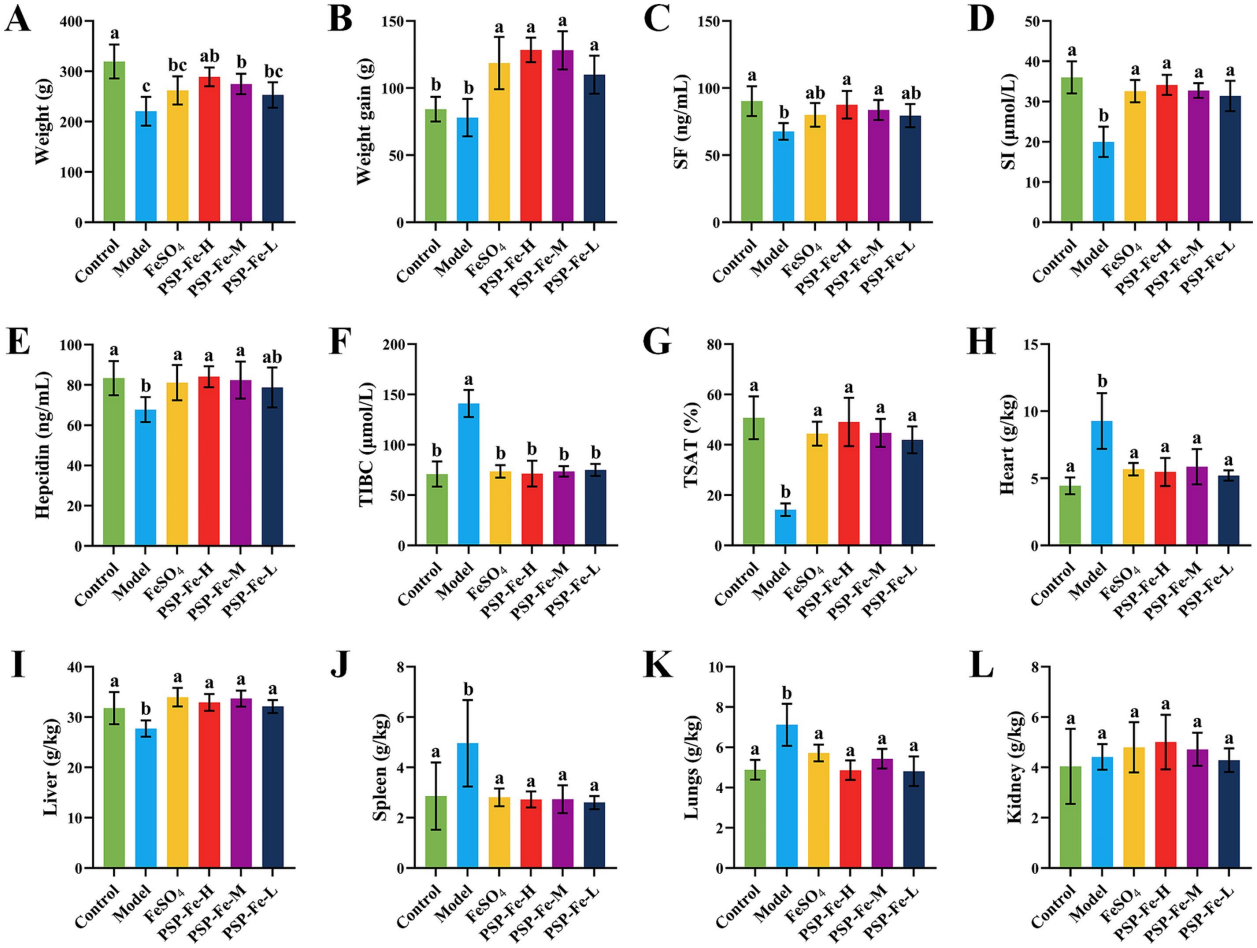
Body weight changes, hematological indicators and organ coefficients in rats fed a normal diet and in IDA rats treated with different iron supplements. **(A)** Body weight. **(B)** Body weight gain. **(C)** Serum ferritin. **(D)** Serum iron. **(E)** Hepcidin. **(F)** Total iron binding capacity. **(G)** Transferrin saturation. **(H)** Heart coefficient. **(I)** Liver coefficient. **(J)** Spleen coefficient. **(K)** Lungs coefficient. **(L)** Kidney coefficient. Different letters (a–c) showed significant difference (*p* < 0.05).

Figures 2A,B have shown the final body weight and weight gain of the rats in each group. At the end of day 21, the weights of Control, PSP-Fe-H and PSP-Fe-M groups were significantly higher than those of the Model group (*p* < 0.05). Although the mean weights of the PSP-Fe-L and FeSO_4_ groups were higher than those of the Model group, there were no significant differences (*p* > 0.05). The weight of the PSP-Fe-H group was not significantly different from that of the Control group (*p* < 0.05), and there were no significant differences in weights between each PSP-Fe and FeSO_4_ groups (*p* < 0.05). The weight gains of each PSP-Fe and FeSO_4_ groups were significantly higher than that of the Model and Control groups (*p* < 0.05), while there was no significant difference between the Model and Control groups (*p* > 0.05).

#### 3.2.2 Hematological tests

Table 3 and Figures 2C–G have shown the hematological levels of the rats in each group. The levels of HGB, RBC, HCT, MCV, MCH, MCHC, SF, SI, hepcidin and TSAT in the Model group were significantly lower than those in the Control group (*p* < 0.05), while the levels of RDW and TIBC were significantly higher than those in the Control group (*p* < 0.05). After 21 days of gavage with iron supplementation, the levels of HGB, RBC and HCT in the PSP-Fe-H and PSP-Fe-M groups were normalized (*p* > 0.05 versus the Control group). While the levels of HGB and HCT in the FeSO_4_ group were incomplete recovered (*p* < 0.05 versus the Control group). In addition, the levels of MCV, MCH, MCHC and RDW in the PSP-Fe-H group, as well as the level of MCHC in the PSP-Fe-M and FeSO_4_ groups were all recovered (*p* > 0.05 versus the Control group). The level of MCV in the PSP-Fe-M and FeSO_4_ groups were incomplete recovered (*p* < 0.05 versus the Control group), and there was no significant difference between them (*p* > 0.05). The levels of MCH and RDW in the PSP-Fe-M and FeSO_4_ groups were incomplete recovered (*p* < 0.05 versus the Control group), and there were significant difference between them (*p* < 0.05). The levels of SF, SI, hepcidin, TIBC and TSAT in each PSP-Fe and FeSO_4_ groups were all recovered (*p* > 0.05 versus the Control group). These results indicated that an effective dose of PSP-Fe can effectively improve various hematological parameters in IDA rats, showing relatively excellent hematopoietic capacity. Similar pharmacodynamic results were found for oat peptide-ferrous chelate (23).

**Table 3:** Hematological parameters of rats fed standard diet and IDA rats treated with different iron supplements.

Group	N	HGB (g/L)	RBC (10^12^/L)	HCT (%)	MCV (fL)	MCH (pg)	MCHC (g/L)	RDW (%)
Control	7	148.57 ± 7.09^a^	7.27 ± 0.40^a^	45.90 ± 2.74^a^	61.84 ± 1.32^a^	20.41 ± 0.59^a^	330.71 ± 6.50^a^	14.56 ± 0.54^d^
Model	7	90.86 ± 3.53^c^	4.32 ± 0.73^b^	25.59 ± 3.02^c^	44.19 ± 1.95^d^	16.64 ± 0.74^c^	307.29 ± 6.78^b^	25.73 ± 1.02^a^
FeSO_4_	7	134.14 ± 7.13^b^	7.06 ± 0.30^a^	40.44 ± 2.40^b^	55.46 ± 3.22^c^	17.29 ± 0.79^c^	323.43 ± 8.72^a^	18.54 ± 0.84^b^
PCP-Fe-H	7	143.57 ± 6.60^ab^	7.17 ± 0.38^a^	43.87 ± 2.69^ab^	59.61 ± 1.90^ab^	19.67 ± 0.66^a^	329.86 ± 7.47^a^	15.44 ± 0.80^cd^
PCP-Fe-M	7	141.14 ± 5.21^ab^	7.07 ± 0.49^a^	42.64 ± 2.93^ab^	57.71 ± 2.29^bc^	18.47 ± 0.64^b^	324.57 ± 7.41^a^	16.63 ± 0.72^c^
PCP-Fe-L	7	135.29 ± 6.18^b^	7.01 ± 0.24^a^	40.49 ± 2.19^b^	55.04 ± 2.47^c^	17.53 ± 0.50^bc^	322.86 ± 2.79^a^	18.40 ± 0.55^b^

Different letters (a–d) showed significant difference (*p* < 0.05).

#### 3.2.3 Organ coefficient

Figures 2H–L demonstrated the organ coefficients of rats in each group. The heart coefficient, lungs coefficient, and spleen coefficient of Model group were significantly higher than those of the Control group (*p* < 0.05). After treatment with iron supplements, the above coefficients of each PSP-Fe and FeSO_4_ groups were all recovered (*p* > 0.05 versus the Control group). Kidney coefficient were not significantly different between all groups (*p* > 0.05).

#### 3.2.4 Histological analysis

**Figure 3:**
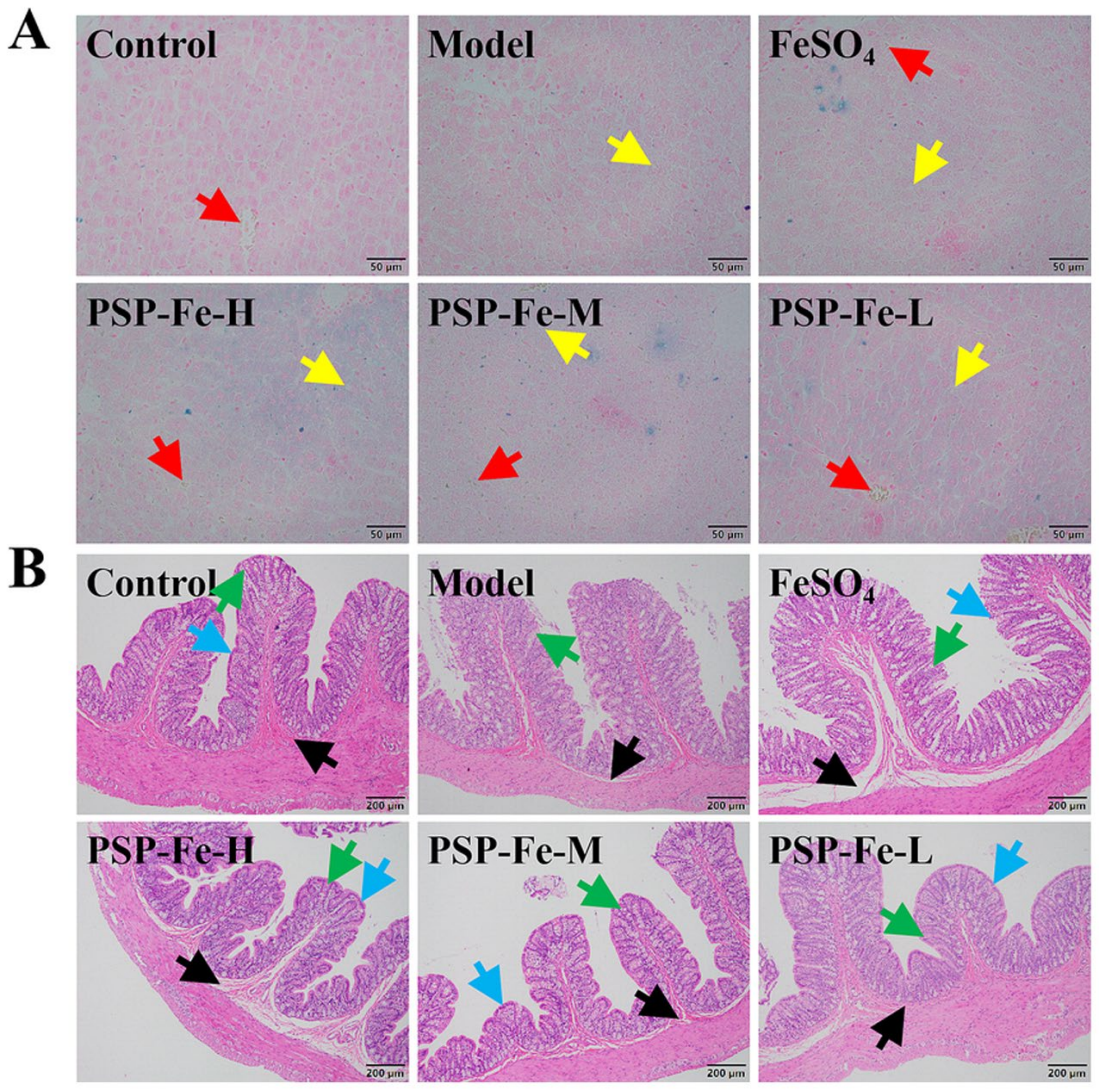
Organ tissue sections from rats. **(A)** Liver. **(B)** Colon. Different colored arrows point toward representative tissue counterparts. Red: hemosiderin; yellow: lipid droplets; green: goblet cell; blue: colonic villi; black: lamina propria.

Prussian blue staining of the liver and H&E staining of the colon in each group of rats have shown in Figures 3A,B. Hemosiderin deposition could be clearly observed in liver sections of Control, each PSP-Fe and FeSO_4_ groups, whereas the presence of hemosiderin deposition was difficult to observe in the Model group. Lipid droplets were found in the liver sections of all groups except Control group. The villi of the colon were significantly damaged in the Model and FeSO_4_ groups. In contrast, the Control and each PSP-Fe groups had intact colonic villi with abundant numbers of goblet cell.

### 3.3 High-throughput sequencing analysis of the gut microbiota

#### 3.3.1 Endemic species analysis

**Figure 4:**
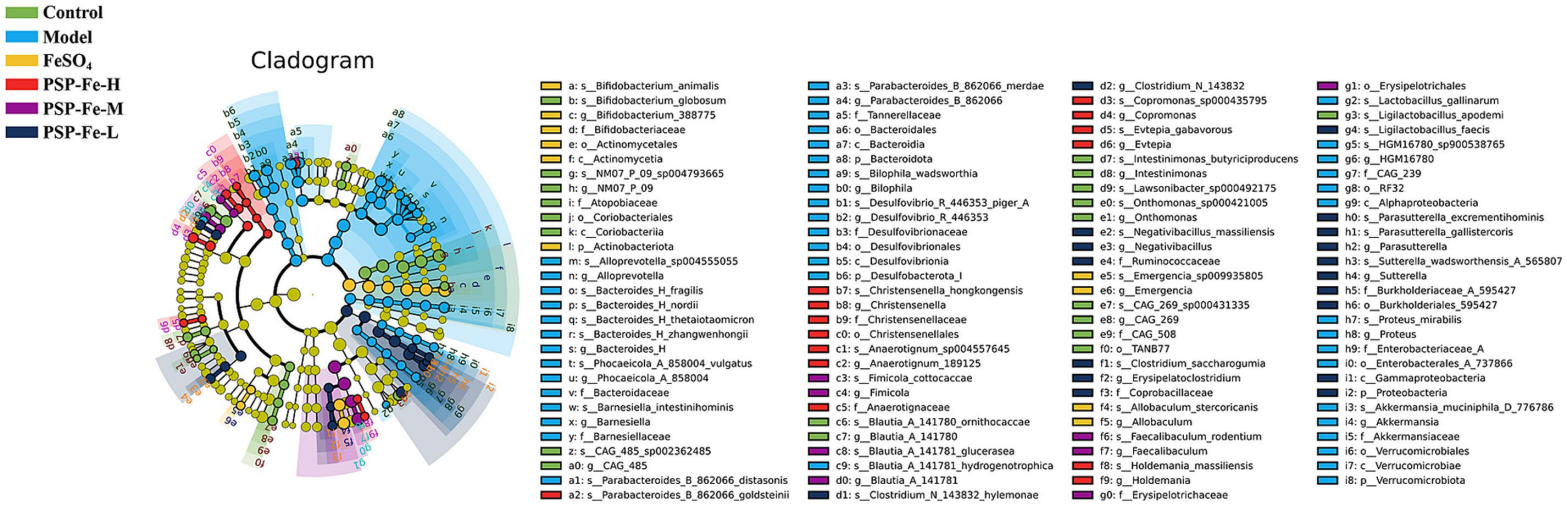
LEfSe analysis (LDA significant threshold >2.0).

**Figure 5:**
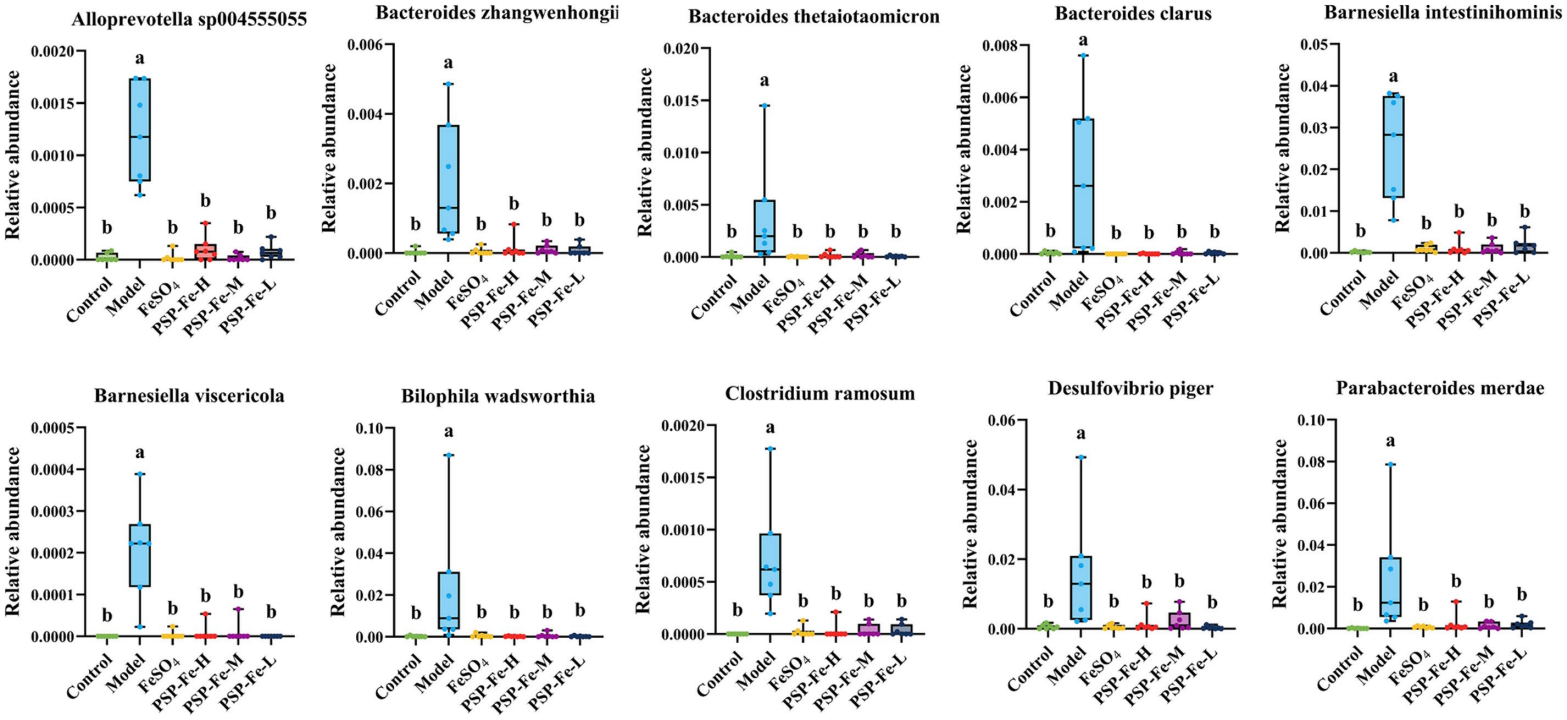
Ten statistically significant microbial markers in the Model group. Different letters (a,b) showed significant difference (*p* < 0.05).

Linear discriminant effect size (LEfSe) analysis was used to identify microbial markers that were significant in the Model group (Figure 4). Ten statistically significant microbial markers were finally identified (Figure 5). *Alloprevotella sp004555055*, *Bacteroides zhangwenhongii*, *Bacteroides thetaiotaomicron*, *Bacteroides clarus*, *Barnesiella intestinihominis*, *Barnesiella viscericola*, *Bilophila wadsworthia*, *Clostridium ramosum*, *Desulfovibrio piger*, and *Parabacteroides merdae* had a significantly higher relative abundance in the Model group than in the other groups (*p* < 0.05). Currently, there are no reported associations between these microbial markers and IDA.

#### 3.3.2 Metabolic pathway enrichment analysis

**Figure 6:**
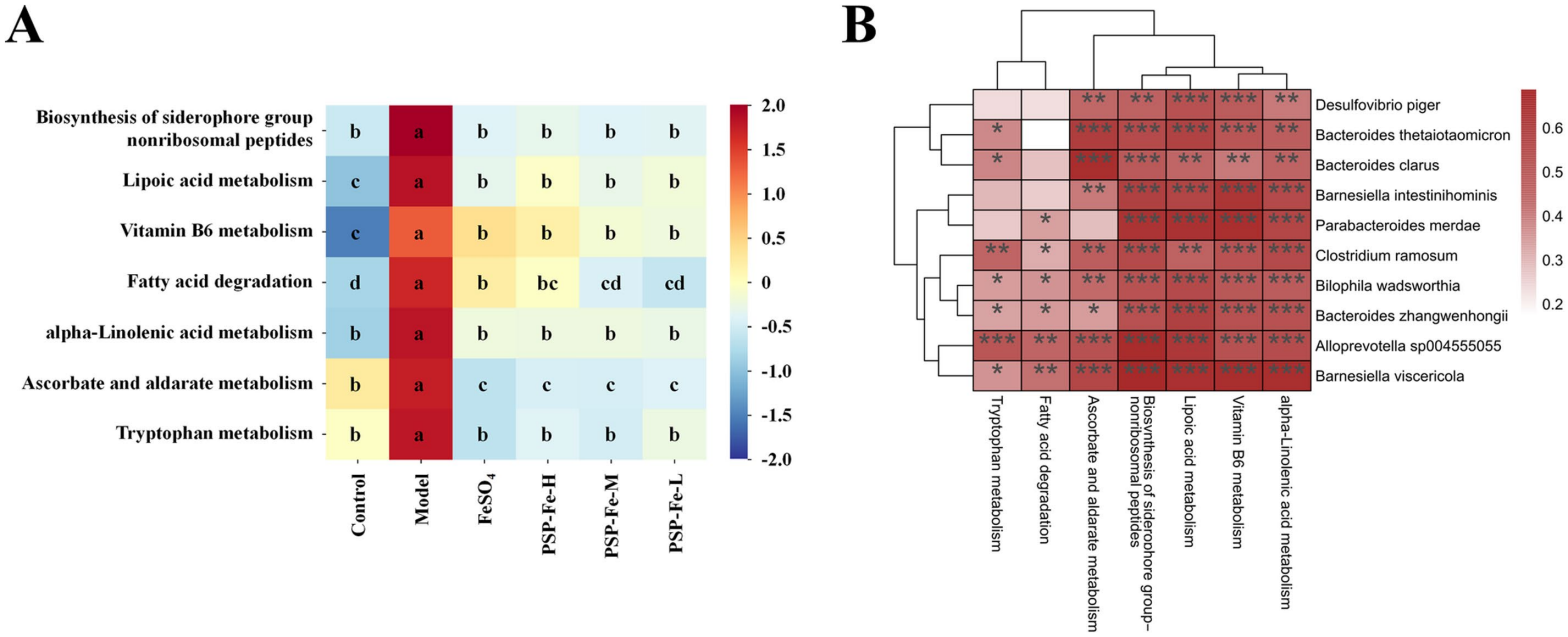
**(A)** Heatmap of metabolic pathway enrichment analysis. **(B)** Spearman correlation analysis between 10 microbial markers and metabolic pathways. ^*^p < 0.05, ^**^p < 0.01, and ^***^p < 0.001. Different letters (a–d) showed significant difference (*p* < 0.05).

The metabolic pathway enrichment analysis has shown in Figure 6A. The biosynthesis of siderophore group nonribosomal peptides, vitamin B6 metabolism, lipoic acid metabolism, fatty acid degradation, α-linolenic acid metabolism, ascorbate metabolism and tryptophan metabolism were all significantly upregulated in the Model group compared to the other groups (*p* < 0.05).

The Spearman correlation analysis between the 10 microbial markers and metabolic pathways has shown in Figure 6B. All microbial markers were significantly positively correlated with the biosynthesis of siderophore group nonribosomal peptides, VB6 metabolism, and lipoic acid metabolism. All microbial markers showed a significant positive correlation with ascorbate metabolism, except *P. merdae*; and showed a significant positive correlation with tryptophan metabolism, except *B. intestinihominis*, *D. piger*, and *P. merdae*. In addition, all microbial markers were significantly positively correlated with α-linolenic acid metabolism, and six microbial markers (*A*. *sp004555055*, *B*. *zhangwenhongii*, *B. viscericola*, *B. wadsworthia*, *C. ramosum*, and *P. merdae*) were significantly positively correlated with fatty acid degradation.

### 3.4 Toxicity evaluation of PSP-Fe *in vitro*

**Figure 7:**
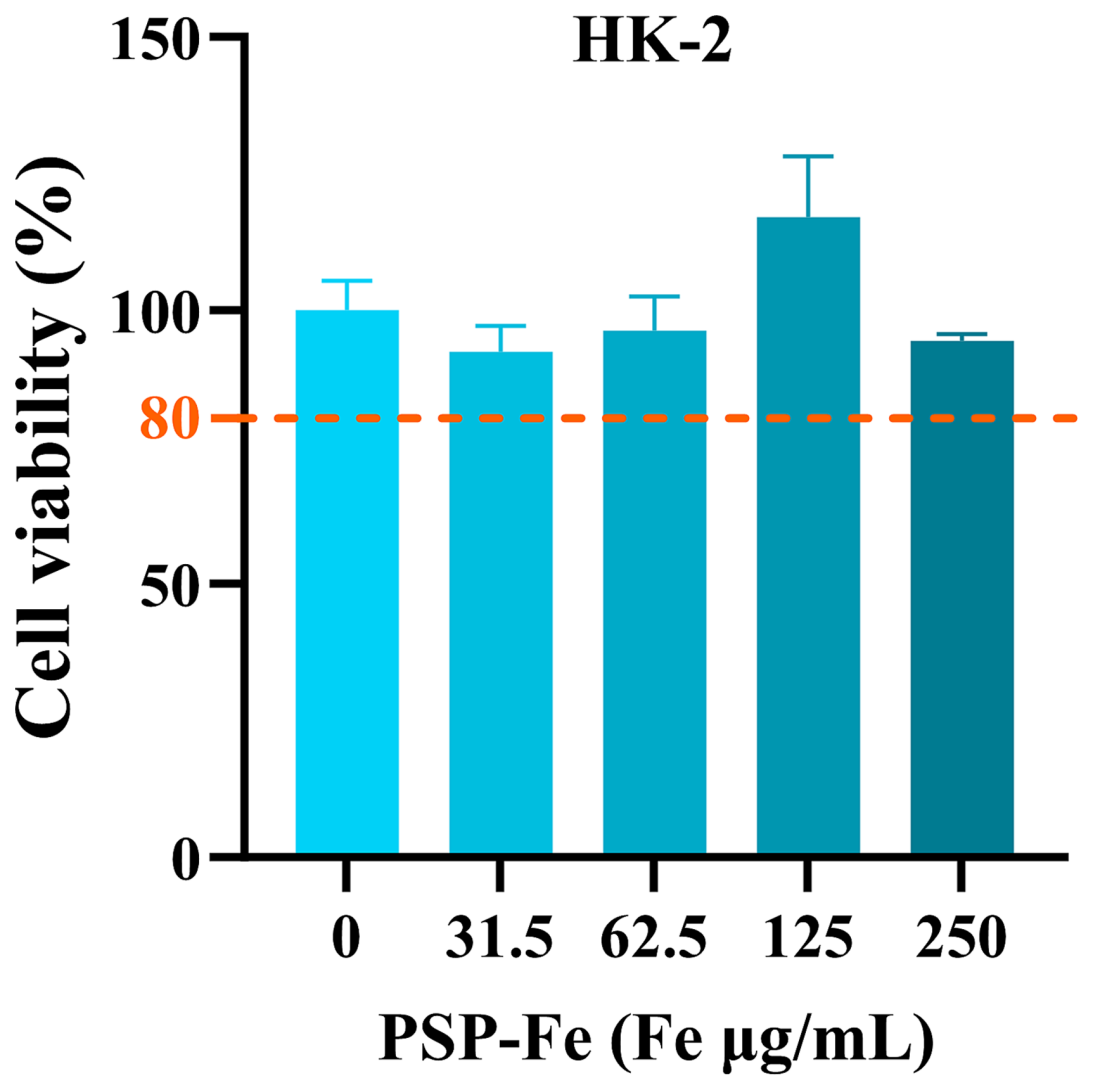
The cell viability of HK-2 treated with different concentrations of PSP-Fe (Fe = 0, 31, 63, 125, and 250 μg/mL).

Figure 7 has shown the cell viability of HK-2 treated with different concentrations of PSP-Fe. The cell viability exceeded when the Fe content was within 250 μg/mL. This results indicated that PSP-Fe had no significant toxicity *in vitro*.

### 3.5 Toxicity evaluation of PSP-Fe *in vivo*

**Figure 8:**
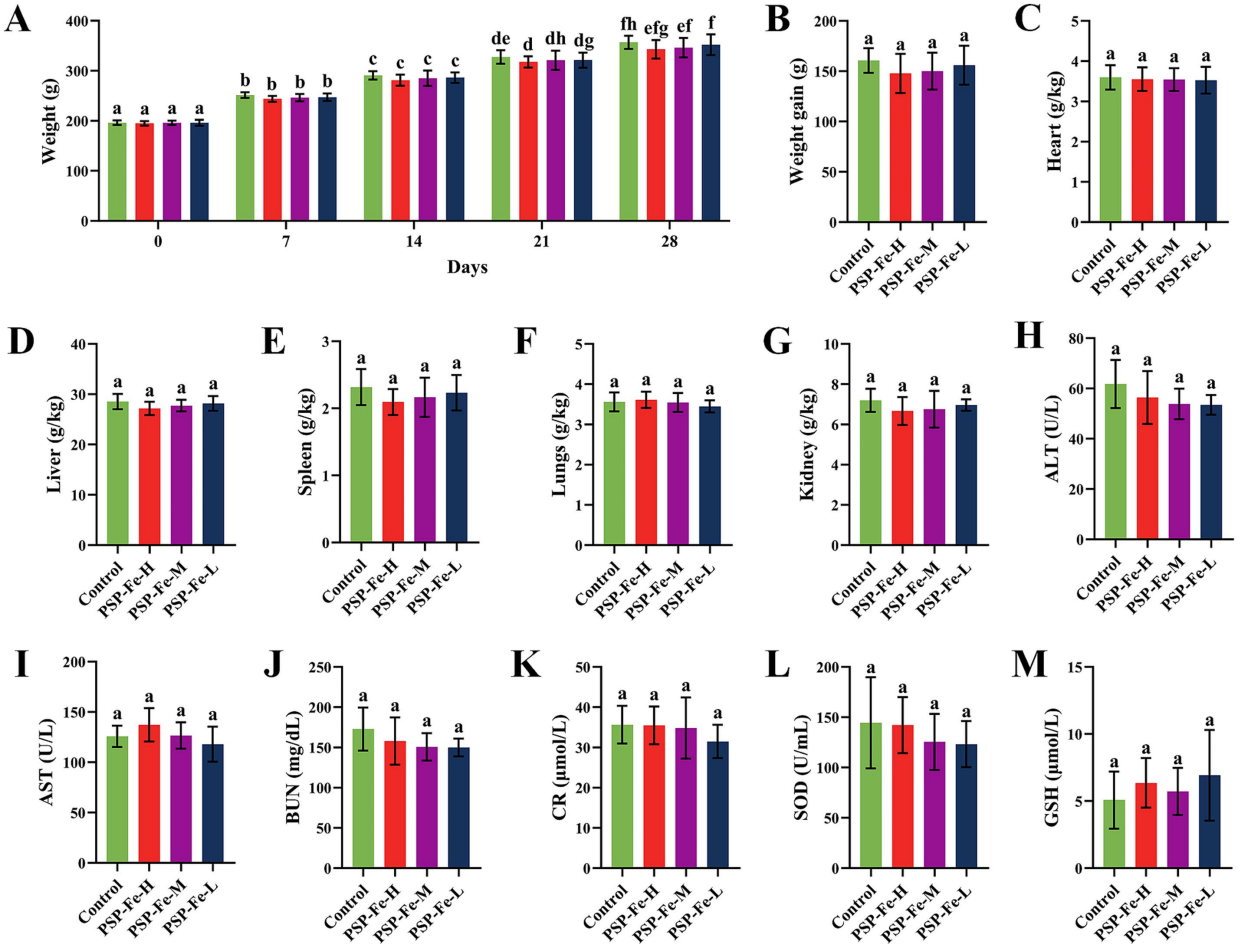
Body weight changes, organ coefficients and hematological indicators in rats fed a normal diet for toxicity evaluation. **(A)** The change of the body weight during the 28-day experiment. **(B)** Body weight gain. **(C)** Heart coefficient. **(D)** Liver coefficient. **(E)** Spleen coefficient. **(F)** Lungs coefficient. **(G)** Kidney coefficient. **(H)** Alanine aminotransferase. **(I)** Aspartate aminotransferase. **(J)** Blood urea nitrogen. **(K)** Creatinine. **(L)** Superoxide dismutase. **(M)** Glutathione. Different letters (a–f) showed significant difference (*p* < 0.05).

**Figure 9:**
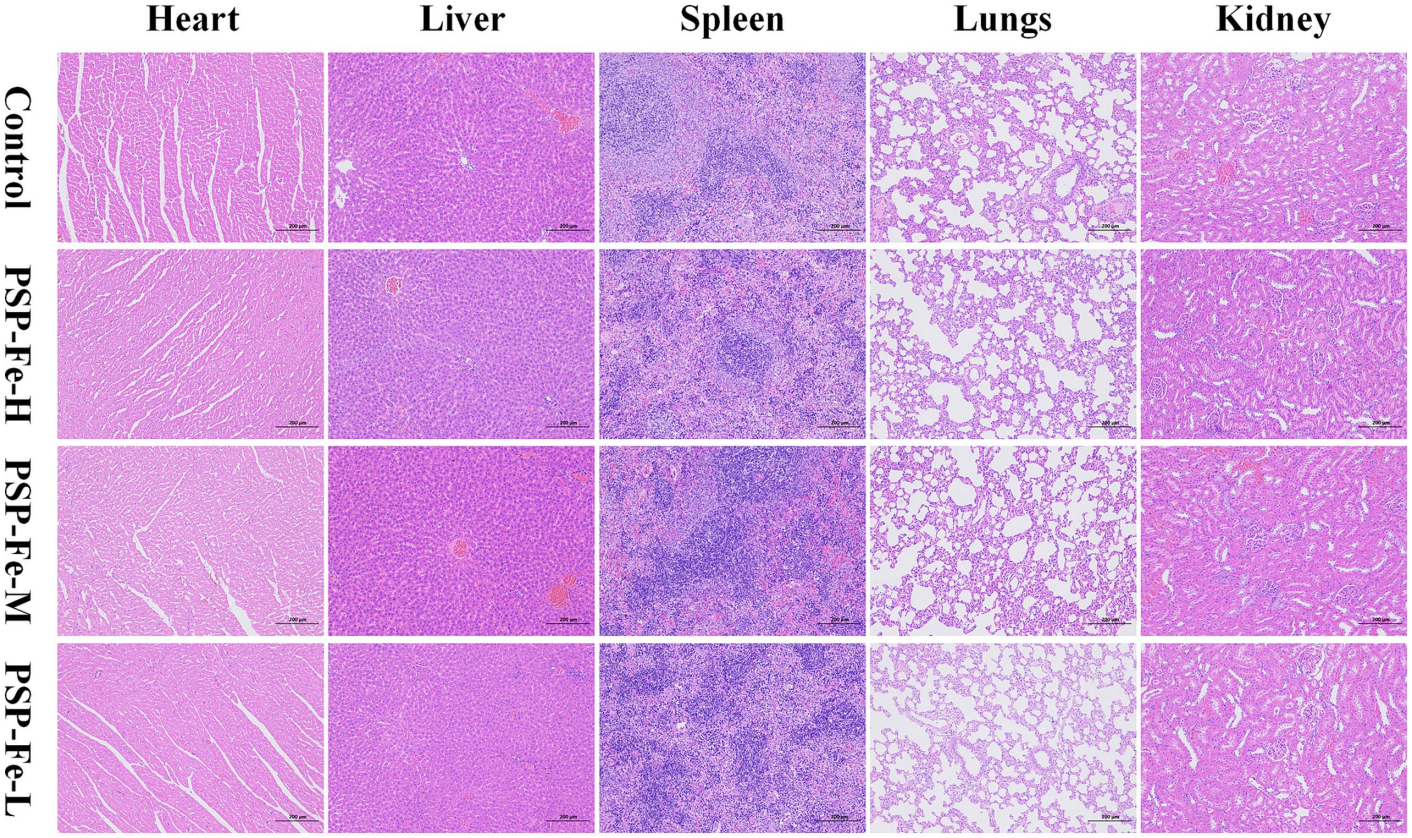
Organ tissue sections from rats for toxicity evaluation.

Figures 8A,B have shown the change of the body weight of the rats in each group during the 28-day experiment. There were no significant differences (*p* > 0.05) in the final body weight and weight gain between the groups. Figures 8C–G demonstrated the organ coefficients of rats in each group. The heart coefficient, liver coefficient, spleen coefficient, lungs coefficient and kidney coefficient have no significant differences (*p* > 0.05) between the groups. Figures 8H–M have shown the hepatotoxicity (ALT and AST), nephrotoxicity (BUN and CR) and antioxidant capacity (SOD and GSH) of rats. There were no significant differences (*p* > 0.05) in these indicators between the groups. H&E staining of the heart, liver, spleen, lungs and kidney in each group of rats have shown in Figure 9. Histological observations of heart, liver, spleen, lungs, and kidney did not reveal significant differences. These results suggested that PSP-Fe had no obvious toxicity *in vivo*.

## 4 Discussion

IDA usually adversely affects the normal growth of the organism. In our study, rats in the Model group showed decreased activity, light brown feces, and slightly pale pupils, ears, and paws compared to the Control group. Importantly, IDA can also cause slow weight gain. Nevertheless, after PSP-Fe treatment, the slow weight gain of the rats was alleviated, pale pupils, ears and paws became reddish, and feces change from brown to black. This indicated that PSP-Fe could alleviate the low weight caused by IDA and had a positive effect on the normal growth of rats.

One of the manifestations of IDA is significantly lower levels of HGB, RBC, HCT, SF, SI, hepcidin, and TSAT in the body. SF is the most sensitive indicator for the clinical diagnosis of IDA (37). Low levels of ferritin are only seen in patients suffering from IDA, no other possible pathology would confound this finding. Other indicators used to supplement the diagnosis may be SI, hepcidin and TSAT. SI is reduced in absolute ID as well as in functional ID or inflammatory conditions. Hepcidin is a peptide hormone produced in the liver that negatively regulates the entry of iron into the bloodstream. The synthesis of peptide hormones is regulated at the transcriptional level controlled by serum iron concentration. Hepcidin expression is upregulated when SI levels are elevated. Hepcidin triggers tyrosine phosphorylation, internalization, and ubiquitin mediated degradation in lysosomes by binding to the ferroportin 1 (FPN1) in tissue duodenal enterocytes, liver cells, Kupffer cells, and other target cells (38). After removing the ferroportin FPN1 from the plasma membrane, the cellular iron output is shut off, resulting in a decrease in SI levels. In addition, high concentrations of iron in the liver, inflammation, and physical activity can also upregulate hepcidin expression, while iron deficiency, erythropoiesis, hypoxia, and endocrine signals (testosterone, estrogen, and growth factors) can downregulate hepcidin expression (8, 39). TSAT is a major indicator and determinant of systemic iron homeostasis. The TSAT is determined by the amount of iron absorbed from the intestine, recycled and released by macrophages, and used for erythropoiesis.

The organ coefficients could reflect, to some extent, the damage and repair effects of the disease or drugs on organs. The heart coefficient, lungs coefficient, and spleen coefficient were significantly higher in the Model group than in the Control and different iron supplementation groups. This may be due to the decrease in the concentration of erythrocytes and HGB in the blood; to meet the body’s normal oxygen demand, compensatory hypertrophy and hyperplasia of the heart and lungs are required to enhance the ability to pump blood and exchange oxygen at the alveoli. This is consistent with the findings of Wang et al. (40). Kuvibidila et al. (41) has demonstrated that ID activates splenocytes and promotes cell proliferation. In addition, IDA was observed to cause damage to the liver. After treatment with PSP-Fe, the heart coefficient, lungs coefficient, spleen coefficient and liver coefficients were normalized. The effect of IDA on the kidney was not significant, at least on the organ coefficient. This is similar to the results of Song et al. in a study on the *Lachnum* YM226 melanin-iron complex (42).

The liver is one of the most important tissues for iron storage. When SI is not used for erythropoiesis, iron will be stored in the liver for later use. Hepatic iron deposition can physiologically range between 300 mg and 1 g. For patients suffering from hereditary hemochromatosis, the hepatic iron deposition can reach up to 25–30 g (43). Due to iron depletion caused by IDA, it was difficult to observe hemosiderin deposition in liver sections in the Model group. After treatment with PSP-Fe, iron storage in the liver was significantly restored. In addition, lipid droplets were found in the liver of all groups except the Control group, suggesting steatosis, and relying solely on iron supplements does not seem to cure it in the short term. This is consistent with the results observed by Pan et al. (44).

Sections of colon tissue were stained with H&E to further investigate the effects of IDA, FeSO_4_, and PSP-Fe on colon morphology. Compared with the Control group, the villi were damaged in the Model group. The impaired intestinal barrier may increase the chance of toxic substances, such as lipopolysaccharides and pathogenic bacteria, entering the tissue through the body’s circulation, resulting in infection. After treatment with PSP-Fe, the intestinal villi were significantly repaired, and the overall structure was more intact. However, FeSO_4_-treated colon tissues were poorly repaired, and damage to the villi remained. Similar results were reported in a study on the Ejiao peptide-iron chelates (45).

Under low iron conditions, some pathogenic bacteria will secrete siderophores to increase the efficiency of iron uptake from the external environment (46). Siderophores are low molecular weight compounds with a high binding affinity for ferric iron. Siderophores can be classified into three main families based on their characteristic functional group: catecholate, hydroxamate, and carboxylate (47). They are generally synthesized by the nonribosomal peptide synthetase or polyketosomal synthetase domains and are secreted by the effervescent pump (48, 49). In the Model group, the biosynthesis of siderophore group nonribosomal peptides was significantly upregulated, suggesting that gut microbes compete with the host for iron sources. This may lead to the persistence of IDA.

Heme, which is involved in the composition of HGB, is synthesized primarily in the young erythrocytes and reticulocytes of the bone marrow. The basic raw materials for the synthesis of heme in the body are glycine, succinyl coenzyme A (succinyl-CoA), and Fe^2+^. In the mitochondria, glycine, and succinyl-CoA are condensed to form δ-amino levulinic acid (ALA), which is catalyzed by ALA synthase. ALA synthase is the rate-limiting enzyme for the synthesis of heme. This reaction requires pyridoxal-5′-phosphate (PLP) as a coenzyme. Pyridoxal and PLP are the main forms of vitamin B6 (VB6) present in the body. In addition, succinyl-CoA is produced by oxidative removal of the carboxyl group from α-ketoglutaric acid by the oxoglutarate dehydrogenase complex, and lipoic acid is one of the essential coenzymes that make up the oxoglutarate dehydrogenase complex (50). VB6 metabolism and lipoic acid metabolism were significantly upregulated in the Model group, which may lead to reduced heme synthesis in the host, which further affects HGB synthesis.

According to previous reports, IDA can lead to lipid metabolism disorders (51–53). The fatty acid degradation and α-linolenic acid metabolism of the gut microbiota in the Model group were significantly upregulated compared to the Control group. Abnormalities in lipid metabolism were reversed after treatment with PSP-Fe. Iron solubility is also a limiting factor in iron absorption because iron ions precipitate readily when transitioning from the low pH environment of the stomach to the high pH environment of the duodenum. Ascorbic acid has reducing properties that allow it to form soluble iron complexes with iron ions to improve iron bioavailability and reduce the opportunity for siderophores to bind to ferric iron (54). However, ascorbate metabolism was upregulated in the Model group.

Interestingly, we found that tryptophan metabolism was also significantly higher in the Model group than in the Control group. Julian reported that in a cohort of 430 patients, 115 patients with ID had lower serum tryptophan levels than 315 patients without ID (55). As a nutritional pyrrole source, tryptophan is also essential for HGB synthesis. And there are six tryptophan residues that located in the α- and β-subunits of human HGB (56). Tryptophan availability limits HGB production, which may be one of the reasons for the positive correlation between HGB concentration and tryptophan. Weiss elucidated a possible process for degrading tryptophan under inflammatory conditions: tryptophan is degraded by cytokine-induced (e.g., interferon-γ, tumor necrosis factor-α) indoleamine 2,3-dioxygenase to kynurenine, which affects erythroid progenitor cell growth and differentiation (57). Under inflammatory conditions, the catabolism of tryptophan to kynurenine is accelerated, resulting in a deficiency of this essential amino acid. Eleftheriadis et al. (58) suggests that kynurenine, by competing with hypoxia-inducible factor 2α, may contribute to anemia of inflammation by decreasing erythropoietin and increasing hepcidin production.

Previous studies have shown that *B. thetaiotaomicron* does not produce siderophores (59). When pathogenic *Salmonella* was present *in vitro* or the inflammatory environment of the mouse intestine, *B. thetaiotaomicron* used siderophores produced by other species to grow under iron-restricted conditions, such as *Escherichia coli* Nissle 1917. Thus, the correlation analysis implied that these microbial markers may prey on iron through siderophores produced by themselves or other species, and also upregulate VB6 metabolism, lipoic acid metabolism, ascorbate metabolism, and tryptophan metabolism to impede HGB synthesis. They may be potential disease markers, therapeutic targets, or treatments for IDA; however, this requires further experimental validation. In addition, these microbial markers also showed significantly positively correlated with α-linolenic acid metabolism and fatty acid degradation, suggesting that they might also be involved in the regulation of lipid metabolism.

In hepatocytes, ALT is mainly distributed in the cytoplasm, while AST is distributed in both the mitochondria and the cytoplasm. When hepatocytes are damaged, AST and ALT are released into the circulatory system, resulting in elevated serum AST and ALT levels (60). BUN is the end product of protein metabolism in the body and is mainly produced by the liver. CR is a compound produced in the process of muscle energy production and is metabolized by creatine phosphate. BUN and CR are excreted in the urine through glomerular filtration, which reflects the renal function (61). As the antioxidant status indices, SOD and GSH are mainly involved in antioxidant processes in organisms (62).

## 5 Conclusion

In summary, the binding modes of PSP to iron were elucidated and the anti-IDA efficacy of PSP-Fe was also evaluated. The biosynthesis of siderophores, as well as the consume of VB6, lipoic acid, ascorbic acid and tryptophan may impede iron absorption and HGB synthesis of host. The low cost, easy availability, and high bioavailability of PSP-Fe make it a potential source of novel functional food for iron supplementation.

## Data Availability

The original contributions presented in the study are included in the article/Supplementary material, further inquiries can be directed to the corresponding author.
